# Occurrence and Molecular Characterization of *Cryptosporidium* spp. in Swine Farms in Northeastern Spain

**DOI:** 10.3390/pathogens14070665

**Published:** 2025-07-05

**Authors:** Laura Garza-Moreno, Celia León, Joaquín Quílez

**Affiliations:** 1 Department of Animal Pathology, Faculty of Veterinary Sciences, University of Zaragoza, 50013 Zaragoza, Spain; 2 Agri-Food Institute of Aragón (IA2), University of Zaragoza-CITA, 50013 Zaragoza, Spain

**Keywords:** *Cryptosporidium* spp., pigs, *Cryptosporidium suis*, *Cryptosporidium scrofarum*, zoonosis

## Abstract

*Cryptosporidium* spp. are protozoan parasites that cause cryptosporidiosis, an enteric disease that can affect a wide range of vertebrate hosts. Pigs play a potential role in the transmission of *Cryptosporidium* spp. to humans, although infections are most often subclinical. This study aimed to assess the occurrence and molecular characterization of *Cryptosporidium* spp. in swine farms located in Aragón, northeastern Spain. Fecal samples (*n* = 72) were collected from 10 breeding farms, encompassing various production stages (lactation, nursery, fattening, and/or wean-to-finish). Data regarding the type of production system (two- or three-stage), production stages, and farming facilities (the type of flooring and water source) associated with the parasite presence were also analyzed using a questionnaire. The results showed that *Cryptosporidium* spp. were more frequently detected in fecal samples originating from three-stage production systems (21.9%) compared to two-stage systems (12.5%). Samples from the fattening stage exhibited the highest positivity rate and estimated oocyst count (3.0 oocyst/microscopic field). Furthermore, the molecular characterization of *Cryptosporidium* spp. revealed the circulation of multiple species both among farms and within the same pig production flow, with *Cryptosporidium scrofarum* being the most prevalent species (7/72; 9.7%), followed by *Cryptosporidium suis* (1/72; 1.4%). These findings underscore the importance of the surveillance and molecular characterization of *Cryptosporidium* spp. for controlling infections in pigs, considering the potential for the zoonotic transmission of this parasite to humans.

## 1. Introduction

*Cryptosporidium* spp. are zoonotic parasites with a global distribution, affecting a wide range of vertebrate hosts, including humans. Infection with this protozoan can lead to cryptosporidiosis, an enteric disease recognized as a significant zoonotic illness worldwide [1]. The main route of transmission of this parasite is fecal–oral, involving the ingestion of infective *Cryptosporidium* spp. oocysts from contaminated environments, food, or water [2].

Among susceptible animals, pigs are considered a significant reservoir host for *Cryptosporidium* spp. [1,2,3,4,5,6]. The global prevalence of *Cryptosporidium* spp. in pigs was estimated to be 16.3% [6]. The highest infection rate of this parasite in pigs per continent was found in Africa (40.8%), followed by Europe (18.3%). Within Europe, the United Kingdom and Ireland reported the highest prevalence (44.6%), while Germany showed the lowest prevalence (0.4%). In Spain, the estimated prevalence of *Cryptosporidium* spp. was 13.7% [4], based on published data from previous studies in domestic pigs [7,8], black Iberian pigs [9], and wild boars [9,10].

In most cases, *Cryptosporidium* spp. infections in pigs are subclinical [11,12]. Nevertheless, in some cases, cryptosporidiosis may present clinical manifestations, particularly in the presence of co-infections with other enteric pathogens such as *Cystoisospora suis* and/or Rotavirus. In those scenarios, pigs may exhibit diarrhea, reduced feed conversion rates, and even mortality [7,13,14,15]. Previous research indicates that the development and severity of clinical signs in pigs and their severity are influenced by factors such as the age of the infected pigs [16], farm management practices [17], and the specific *Cryptosporidium* species or genotype involved [12]. To date, 13 species/genotypes of this protozoan have been identified in pigs, with *Cryptosporidium scrofarum*, *Cryptosporidium suis*, and *Cryptosporidium parvum* being the most prevalent species detected both globally [6] and in Spain [7,8,9]. These three *Cryptosporidium* species have also been detected in humans, highlighting their zoonotic potential [18,19,20,21] and the importance of implementing effective control measures.

The fact that one to ten infective oocysts of *Cryptosporidium* spp. can initiate infection and cause disease [22,23], coupled with the prolonged survival of these oocysts under diverse environmental conditions [24] and the close contact between pigs and humans, indicates a considerably high zoonotic transmission risk of *Cryptosporidium* spp. from pigs [6,25]. Furthermore, the potential for water resource contamination is significant if swine manure is not properly managed [25]. The increasing implementation of intensive swine production systems in Aragón, a region in northeastern Spain that held the largest Spanish pig population in 2024 [26], results in the generation of a significant volume of slurry. Despite this elevated risk and the potential for widespread contamination, current information on the prevalence of *Cryptosporidium* spp. in this area remains limited. This study aimed to investigate the occurrence of *Cryptosporidium* spp. across various swine farms in Aragón and to identify the parasite species involved through molecular characterization.

## 2. Materials and Methods

### 2.1. Farm Selection

Ten commercial breeding farms voluntarily participated in this study. The ten farms were located in the area with the highest density of pig farms in Aragón, in northeastern Spain. These farms encompassed various production stages: lactation (Phase 1, P1), nursery (P2), fattening (P3), and/or nursery-fattening (P2 + P3, also referred to as wean-to-finish, WTF). Participating farms were classified into two production system types: (A) three-phase farms, including the P1, P2, and P3 stages, and (B) two-phase farms, including the P1 and WTF stages. 

### 2.2. Sample Collection and Questionnaire

A total of 72 fecal samples were collected by the responsible veterinarian at selected commercial breeding farms between March and June 2024. Among these selected farms, four farms (40.0%) followed a three-stage production system (P1, P2, P3), and six farms (60.0%) utilized a two-stage production system (P1, WTF). For each farm and stage, three pens of animals (10–12 animals per pen) were randomly selected, and samples were collected. The number of samples collected per farm and production stage is summarized in Table 1. Feces samples were collected from the pen floor using gloves and sterile, single-use materials and placed into separate containers (2–3 containers per farm). Following collection, samples were transported to the laboratory at 4 °C for analysis.

Participating swine veterinarians completed a study-specific questionnaire comprising 13 questions, including 6 closed-ended (e.g., yes/no or multiple choice) and 7 semi-closed-ended questions (Supplementary File S1). The questionnaire requested information regarding the farm (ID, location, size, production system type, stage, and facilities, such as flooring type and water source) and the health status of the sampled animals concerning digestive signs potentially related to *Cryptosporidium* spp. (diarrhea, retarded growth, loss of appetite, vomiting, lethargy, etc.). Questionnaire responses showed that none of the pigs in the sampled pens exhibited diarrhea or other digestive clinical signs. All farms had slatted floors, and the water supply originated from bored wells.

### 2.3. Fecal Oocyst Concentration and Examination by Immunofluorescence

*Cryptosporidium* spp. oocysts in fecal samples were concentrated using the formalin-ethyl acetate sedimentation method, as previously described by Young et al. [27]. The presence of oocysts in each concentrated sample was examined by immunofluorescence (IF) using a commercial Aqua-Glo™ G/C Direct Reagent Only Kit (A100FLR-20X, Waterborne, Inc. (New Orleans, LA, USA)) following the manufacturer’s instructions. An estimated oocyst count (EOC) was also performed by evaluating 25 random microscopic fields. The infection intensity in each sample was categorized at 400× magnification as follows: high (>10 oocysts), mild (10–5 oocysts), minimal (<5 oocysts), and no infection (0 oocysts).

### 2.4. DNA Extraction, Detection and Sequencing

DNA extraction from microscopically positive samples was performed using the FavorPrep™ Stool DNA Isolation Mini Kit (Favorgen Biotech Corp (Pingtung County, Taiwan)) according to the manufacturer’s instructions. The extracted DNA was stored at −20 °C until polymerase chain reactions (PCRs) were conducted.

*Cryptosporidium* spp. were detected and characterized by the nested PCR amplification of the small-subunit (SSU) ribosomal RNA gene, as previously described by Xiao et al. [28], to obtain a PCR product of approximately 800 bp long (depending on the species). Positive samples exhibiting a high amplicon intensity were selected for Sanger sequencing. These selected samples were purified using PCR ExoSAP-IT™ (Thermo Fisher Scientific (Waltham, MA, USA)), following the manufacturer’s instructions, and subsequently sequenced using a 3500 XL Genetic Analyzer (Thermo Fisher Scientific).

### 2.5. DNA Sequence and Phylogenetic Analysis

Sequences were aligned and edited using MEGA v11.0.13 software [29]. The resulting consensus sequences were analyzed and subsequently deposited in the GenBank database under the accession numbers PV583372 to PV583379. Representative sequences of *Cryptosporidium* species/genotypes isolated in pigs, such as *C. parvum*, *C. suis*, and *C. scrofarum*, among others, were retrieved from the NCBI database for phylogenetic analysis. Additionally, a sequence of *Cystoisospora suis* was included as the outgroup sequence. Phylogenetic trees were constructed using MEGA 11 software. These trees were generated based on 1000 bootstrap replicates and included the sequences obtained in this study along with the above-mentioned representative sequences. Neighbor-joining trees were constructed based on evolutionary distances calculated according to the Kimura two-parameter model.

### 2.6. Statistical Analysis

Data concerning the questionnaire responses and *Cryptosporidium* spp. positivity were recorded in a Microsoft Excel^®^ spreadsheet. Qualitative variables, including questionnaire responses (information on farms and health status), IF (categorized as high, mild, minimal, no infection), and PCR results (positive/negative), were compared using the Chi-square (χ^2^) test. All statistical analyses were performed using R software (version 4.0.2, R Core Team, Vienna, Austria), with statistical significance set at *p* < 0.05.

## 3. Results

### 3.1. Positivity of Cryptosporidium spp. by Immunofluorescence and EOC

*Cryptosporidium* spp. were detected by immunofluorescence in 12 out of 72 (16.7%) collected samples originating from five different farms (Table 2). Regarding the type of production system used, three-stage farms exhibited a higher proportion of positive farms (7/32, 21.9%) by immunofluorescence compared to two-stage farms (5/40; 12.5%). When considering all sampled farms and production types, the highest positivity for *Cryptosporidium* spp. by immunofluorescence was observed in the nursery and fattening phases (3/12, 25.0% in each production phase), followed by the WTF phase (4/23, 17.4%) and the lactation phase (2/25, 8.0%). No statistically significant association was found between *Cryptosporidium* spp. positivity and the type of production used.

The highest EOC values were observed in Farm A (8.6 oocysts/field), which corresponded to a mild infection intensity. In contrast, Farms B (0.02 oocysts/field), D (0.27 oocysts/field), F (0.35 oocysts/field), and J (0.16 oocysts/field) exhibited minimal infection levels. Statistically significant differences in EOC values were detected between Farm A and the other monitored farms (B–J) (*p* < 0.05). Regarding the type of production system used, three-stage farms showed higher EOC values (2.16 oocyst/field) compared to two-stage farms (0.12 oocyst/field). However, no statistically significant differences in EOC values were found between the two production types.

Finally, the fattening stage showed the highest mean EOC value (3.0 oocysts/field), followed by the weaning (2.75 oocysts/field), wean-to-finish (0.18 oocysts/field) and lactation (0.04 oocysts/field) stages. Nevertheless, no statistical differences in EOC values were observed among these production stages.

### 3.2. Detection of Cryptosporidium spp. by Nested PCR and Sequencing

**Figure 1:**
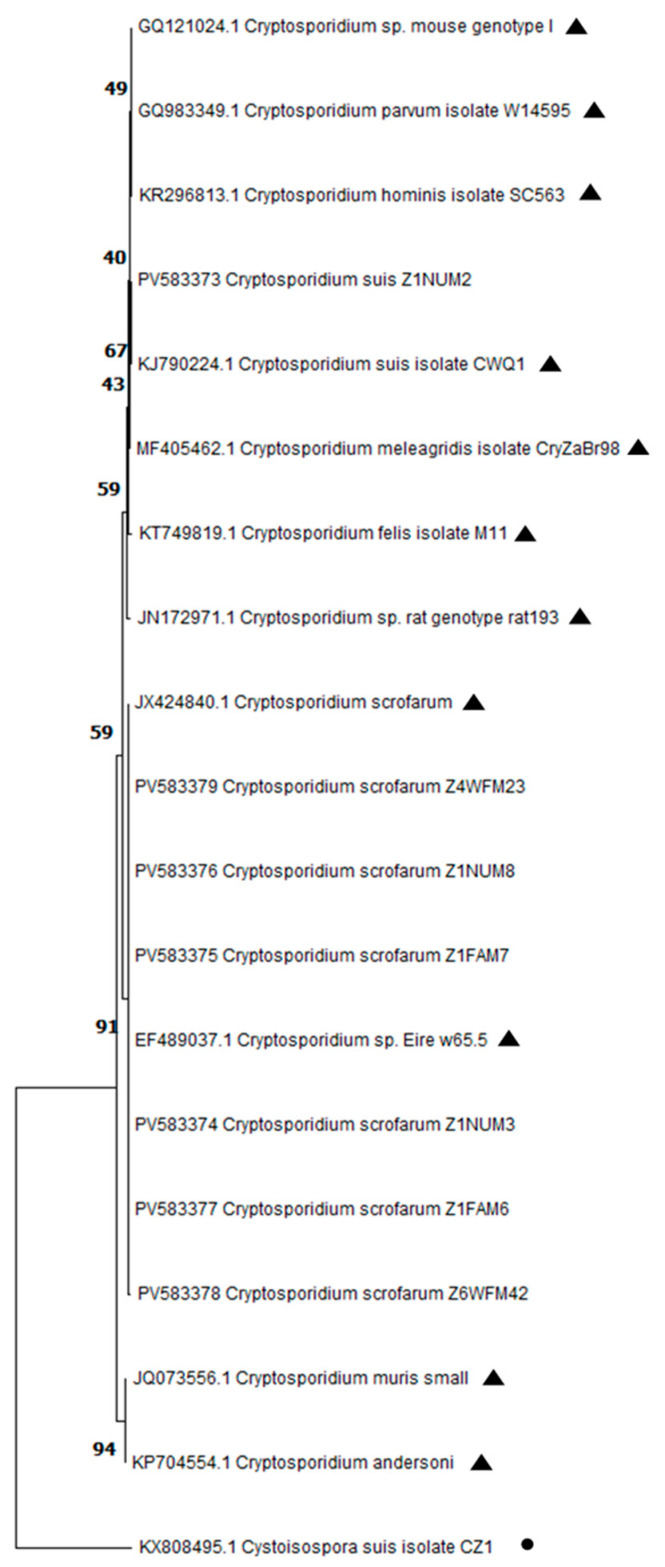
Phylogenetic relationships among *Cryptosporidium* species from this study and reference (▲) and outgroup (●) sequences downloaded from GenBank. A phylogenetic tree based on partial SSU rRNA gene sequences of *Cryptosoporidium* species constructed by the neighbor-joining distance method with 1000 bootstrap replicates using MEGA 11 software.

The twelve samples that tested positive for *Cryptosporidium* spp. by immunofluorescence were subsequently analyzed using nested PCR. Of these, eight samples (8/12, 66.7%) were confirmed positive for *Cryptosporidium* spp. These positive samples corresponded to Farm A (*n* = 6; three from weaning and three from fattening stages), Farm D (*n* = 1; WTF stage), and Farm F (*n* = 1; WTF stage). No amplification was observed in the remaining four samples. The sequencing of *Cryptosporidium* spp.-positive samples revealed that the most prevalent species detected in the tested farms was *C. scrofarum* (7/8), followed by *C. suis* (1/8). The phylogenetic relationships between *Cryptosporidium* species are detailed in Figure 1.

## 4. Discussion

This study investigated the occurrence of *Cryptosporidium* spp. in pigs across different production stages (lactation, nursery, and fattening) and consequently at different ages in various swine farms located in Aragón, northeastern Spain. The results obtained in this study revealed lower prevalence rates of *Cryptosporidium* spp. compared to previous investigations conducted in the same geographical area, where detection rates ranged from 78 to 62% in farms and from 32 to 22% in individual pigs [7,8]. These differences could be attributed to the number of samples analyzed, the age of the animals sampled, and the inclusion of a wean-to-finish production system not considered in earlier studies.

*Cryptosporidium* spp. were most frequently detected on farms following a three-stage production system (21.9%), with the fattening period exhibiting the highest number of positive farms and estimated oocyst count (EOC) values. This finding contrasts with previous studies that reported a higher *Cryptosporidium* spp. prevalence in the post-weaning period (≈25.8%) compared to pre-weaned and fattening pigs [6,7,8,30]. Notably, none of the pigs monitored in this study exhibited diarrhea or other clinical signs potentially associated with *Cryptosporidium* spp. infections during sampling. These findings align with previous studies indicating that *Cryptosporidium* spp. infections are primarily subclinical in pigs, suggesting the potential adaptation of this pathogen to swine [11,12]. This possible adaptation underscores the role of pigs as potential hosts in zoonotic cryptosporidiosis, thus necessitating further research on the prevalence and control of this pathogen in swine populations.

The molecular characterization of *Cryptosporidium* species using SSU rRNA sequence analysis provided valuable data, confirming the presence of different species among the tested farms. Specifically, the most prevalent *Cryptosporidium* species was *C. scrofarum*, which is detected in fecal samples originating from different farms, production types, and stages, while *C. suis* was only reported in a single specimen. These findings are consistent with previous studies conducted at European and global levels, where *C. scrofarum* was also the major species detected in pigs [6,25]. In Spain, a previous study conducted in the same region as the present study also identified *C. scrofarum* (64.0%) and *C. suis* (36.0%) as the most prevalent species in pigs [8]. Notably, in this study, both species *C. scrofarum* and *C. suis* were detected in samples from nursery pigs originating from Farm A. This result confirms that multiple species can co-circulate within a single farm. Such a scenario might imply the potential risk of genetic recombination within species, leading to the emergence of novel subpopulations with unknown virulence and/or drug resistance [31,32,33]. Indeed, mixed infections with the same or different species can lead to recombination events within species, such as the discovery of novel subclades, demonstrating the role of admixture in shaping population structure [33]. Nevertheless, it is important to note that shorter fragments of the expected amplicon were obtained, limiting the accuracy of genotype identification for some sequences. This finding may be explained by the lower quality of sequences obtained from pig samples compared to those from other species (e.g., calves). This could be due to the lower number of oocysts in samples, as well as the potential presence of PCR inhibitors in pig feces. In fact, 4 out of 12 samples that tested positive by immunofluorescence resulted in a negative PCR, supporting the hypothesis of a low number of oocysts in pig samples. Further research is needed to improve sequencing protocols and to evaluate genetic diversity and the potential risks of recombination.

Human cryptosporidiosis is primarily caused by *C. hominis* and *C. parvum*, with the latter being the most significant zoonotic *Cryptosporidium* species [34]. In this study, *C. parvum* was not detected, which is a result consistent with the above-mentioned study conducted in the same geographical area by Suárez-Luengas et al. [8]. These findings strengthen the hypothesis that pigs are not a significant reservoir for human infections with *C. parvum* in this region. Nevertheless, it is worth mentioning that both *C. scrofarum* and *C. suis* have also been sporadically detected in human cases [18,19,20,21]. Given the potential zoonotic risk of *Cryptosporidium* spp. infections, the implementation of stringent internal biosecurity protocols, and proper hygiene practices among farm personnel is crucial to prevent the transmission of this parasite to humans and its spread within farms. These protocols should include thorough hand sanitation for farm workers, the use of gloves when handling animals and/or materials (e.g., crate separators contaminated with feces), and the changing of clothing, among other measures. Similarly, rigorous cleaning and disinfection protocols, especially for slatted floors, are essential to reduce and prevent oocyst environmental contamination and infectious pressure within farms [35,36]. Another critical factor in the spread of *Cryptosporidium* spp. is the contamination of drinking water with oocysts [36,37]. In this study, the impact of floor type and the source of drinking water could not be evaluated since all farms utilized slatted floors and bored wells. Therefore, further research exploring the significance of water sources and farm facilities as risk factors is required to fully elucidate the transmission dynamics of *Cryptosporidium* spp. among pigs and their potential for environmental contamination.

## 5. Conclusions

This study confirmed the presence and genetic diversity of *Cryptosporidium* spp. in pigs across different production stages and farms in Aragón, northeastern Spain. The results indicated that *Cryptosporidium* spp. were detected throughout the pig production flow, with the highest prevalence observed in the three-stage production system and during the fattening stage. The molecular characterization of *Cryptosporidium* spp. confirmed the presence of different species both among and within farms, with *C. scrofarum* being the most prevalent species identified. Given that *Cryptosporidium* spp. is a zoonotic pathogen and considering the predominantly subclinical nature of infections observed in pigs, continuous epidemiological surveillance and the promotion of rigorous hygiene practices among farm personnel are essential measures. Further research focused on elucidating the complex dynamics of *Cryptosporidium* spp. infections in pigs is needed to effectively prevent zoonotic transmission between pigs and humans.

## Figures and Tables

**Table 1:** Number and percentages of collected samples per farm and production stage.

Production Type	Number of Farms (%)	Number of Samples per Production Stage				
		Lactation-P1	Nursery-P2	Fattening-P3	Wean-to-Finish-WTF	Total (%)
3-stages	4	10	23	12	NA	32 (44.4%)
2-stages	6	15	NA	NA	23	40 (55.6%)
**Total**	**10**	**25**	**12**	**12**	**23**	**72 (100%)**

NA: not applicable.

**Table 2:** Number of positive samples for *Cryptosporidium* spp. by immunofluorescence per production stage.

Farm	Production Type	N° Collected Samples	Positive Samples (%)	Number of Samples per Production Stage			
				Lactation-P1	Nursery-P2	Fattening-P3	Wean-to-Finish-WTF
A	3-stages	8	6 (75%) ^a^	-	3	3	NA
B	3-stages	7	1 (14.3%) ^b^	1	-	-	NA
D	2-stages	5	2 (40.0%) ^b^	-	NA	NA	2
F	2-stages	6	2 (33.3%) ^b^	-	NA	NA	2
J	2-stages	6	1 (16.7%) ^b^	1	NA	NA	-
**Total**		**32**	**12 (37.5%)**	**2**	**3**	**3**	**4**

NA: not applicable; different superscript letters indicate statistical significance (*p* < 0.05).

## Data Availability

The complete dataset for this study can be found in Supplementary File S1.

## Supplementary Materials

The following supporting information can be downloaded at: https://www.mdpi.com/article/10.3390/pathogens14070665/s1.

## References

[1] Ryan U., Fayer R., Xiao L. (2014). *Cryptosporidium* species in humans and animals: Current understanding and research needs. Parasitology.

[2] Baldursson S., Karanis P. (2011). Waterborne transmission of protozoan parasites: Review of worldwide outbreaks—An update 2004–2010. Water Res..

[3] Qi M., Zhang Q., Xu C., Zhang Y., Xing J., Tao D., Li J., Zhang L. (2020). Prevalence and molecular characterization of *Cryptosporidium* spp. in pigs in Xinjiang, China. Acta Trop..

[4] Lin Q., Wang X.Y., Chen J.W., Ding L., Zhao G.H. (2015). *Cryptosporidium suis* infection in post-weaned and adult pigs in Shaanxi province, Northwestern China. Korean J. Parasitol..

[5] Feng S., Jia T., Huang J., Fan Y., Chang H., Han S., He H. (2020). Identification of Enterocytozoon bieneusi and *Cryptosporidium* spp. in farmed wild boars (*Sus scrofa*) in Beijing, China. Infect. Genet. Evol..

[6] Chen Y., Qin H., Wu Y., Xu H., Huang J., Li J., Zhang L. (2023). Global prevalence of *Cryptosporidium* spp. in pigs: A systematic review and meta-analysis. Parasitology.

[7] Quílez J., Sánchez-Acedo C., Clavel A., del Cacho E., López-Bernad F. (1996). Prevalence of *Cryptosporidium* infections in pigs in Aragón (northeastern Spain). Vet. Parasitol..

[8] Suárez-Luengas L., Clavel A., Quílez J., Goñi-Cepero M.P., Torres E., Sánchez-Acedo C., del Cacho E. (2007). Molecular characterization of *Cryptosporidium* isolates from pigs in Zaragoza (northeastern Spain). Vet. Parasitol..

[9] Rivero-Juarez A., Dashti A., López-López P., Muadica A.S., Risalde M.L.A., Köster P.C., Machuca I., Bailo B., de Mingo M.H., Dacal E. (2020). Protist enteroparasites in wild boar (*Sus scrofa ferus*) and black Iberian pig (*Sus scrofa domesticus*) in southern Spain: A protective effect on hepatitis E acquisition?. Parasites Vectors.

[10] García-Presedo I., Pedraza-Díaz S., González-Warleta M., Mezo M., Gómez-Bautista M., Ortega-Mora L.M., Castro-Hermida J.A. (2013). Presence of *Cryptosporidium* scrofarum, C. suis and C. parvum subtypes IIaA16G2R1 and IIaA13G1R1 in Eurasian wild boars (*Sus scrofa*). Vet. Parasitol..

[11] Ryan U.M., Samarasinghe B., Read C., Buddle J.R., Robertson I.D., Thompson R.C. (2003). Identification of a novel *Cryptosporidium* genotype in pigs. Appl. Environ. Microbiol..

[12] Vítovec J., Hamadejová K., Landová L., Kvác M., Kvetonová D., Sak B. (2006). Prevalence and pathogenicity of *Cryptosporidium* suis in pre- and post-weaned pigs. J. Vet. Med. B Infect. Dis. Vet. Public Health.

[13] Enemark H.L., Ahrens P., Bille-Hansen V., Heegaard P.M., Vigre H., Thamsborg S.M., Lind P. (2003). *Cryptosporidium* parvum: Infectivity and pathogenicity of the ‘porcine’ genotype. Parasitology.

[14] Hamnes I.S., Gjerde B.K., Forberg T., Robertson L.J. (2007). Occurrence of *Cryptosporidium* and *Giardia* in suckling piglets in Norway. Vet. Parasitol..

[15] Schubnell F., von Ah S., Graage R., Sydler T., Sidler X., Hadorn D., Basso W. (2016). Occurrence, clinical involvement and zoonotic potential of endoparasites infecting Swiss pigs. Parasitol. Int..

[16] Maddox-Hyttel C., Langkjaer R.B., Enemark H.L., Vigre H. (2006). *Cryptosporidium* and Giardia in different age groups of Danish cattle and pigs: Occurrence and management-associated risk factors. Vet. Parasitol..

[17] Němejc K., Sak B., Květoňová D., Hanzal V., Janiszewski P., Forejtek P., Rajský D., Ravaszová P., McEvoy J., Kváč M. (2013). *Cryptosporidium* suis and *Cryptosporidium* scrofarum in Eurasian wild boars (*Sus scrofa*) in Central Europe. Vet. Parasitol..

[18] Kvác M., Kvetonová D., Sak B., Ditrich O. (2009). *Cryptosporidium* pig genotype II in immunocompetent man. Emerg. Infect. Dis..

[19] Sone B., Ambe L.A., Ampama M.N., Ajohkoh C., Che D., Nguinkal J.A., Taubert A., Hermosilla C., Kamena F. (2025). Prevalence and molecular characterization of *Cryptosporidium* species in diarrheic children in Cameroon. Pathogens.

[20] Ahmadpour E., Safarpour H., Xiao L., Zarean M., Hatam-Nahavandi K., Barac A., Picot S., Rahimi M.T., Rubino S., Mahami-Oskouei M. (2020). *Cryptosporidiosis* in HIV-positive patients and related risk factors: A systematic review and meta-analysis. Parasite.

[21] Williams S.V., Matthews E., Inns T., Roberts C., Matizanadzo J., Cleary P., Elson R., Williams C.J., Jarratt R., Chalmers R.M. (2025). Retrospective case-case study investigation of a significant increase in *Cryptosporidium* spp. in England and Wales, August to September 2023. Euro Surveill..

[22] Okhuysen P.C., Chappell C.L., Crabb J.H., Sterling C.R., DuPont H.L. (1999). Virulence of three distinct *Cryptosporidium* parvum isolates for healthy adults. J. Infect. Dis..

[23] Chappell C.L., Okhuysen P.C., Langer-Curry R., Widmer G., Akiyoshi D.E., Tanriverdi S., Tzipori S. (2006). *Cryptosporidium* hominis: Experimental challenge of healthy adults. Am. J. Trop. Med. Hyg..

[24] Alum A., Absar I.M., Asaad H., Rubino J.R., Ijaz M.K. (2014). Impact of environmental conditions on the survival of *Cryptosporidium* and *Giardia* on environmental surfaces. Interdiscip. Perspect. Infect. Dis..

[25] Zahedi A., Ryan U. (2020). *Cryptosporidium*—An update with an emphasis on foodborne and waterborne transmission. Res. Vet. Sci..

[26] Ministerio de Agricultura, Pesca y Alimentación (MAPA) (2023). Resultados De Las Encuestas De Ganado Porcino.

[27] Young K.H., Bullock S.L., Melvin D.M., Spruill C.L. (1979). Ethyl acetate as a substitute for diethyl ether in the formalin-ether sedimentation technique. J. Clin. Microbiol..

[28] Xiao L., Escalante L., Yang C., Sulaiman I., Escalante A.A., Montali R.J., Fayer R., Lal A.A. (1999). Phylogenetic analysis of *Cryptosporidium* parasites based on the small-subunit rRNA gene locus. Appl. Environ. Microbiol..

[29] Tamura K., Stecher G., Kumar S. (2021). MEGA 11: Molecular evolutionary genetics analysis version 11. Mol. Biol. Evol..

[30] Izumiyama S., Furukawa I., Kuroki T., Yamai S., Sugiyama H., Yagita K., Endo T. (2001). Prevalence of *Cryptosporidium parvum* infections in weaned piglets and fattening porkers in Kanagawa Prefecture, Japan. Jpn. J. Infect. Dis..

[31] Wang N., Wang K., Liu Y., Zhang X., Zhao J., Zhang S., Zhang L. (2022). Molecular characterization of *Cryptosporidium* spp., *Enterocytozoon bieneusi* and *Giardia duodenalis* in laboratory rodents in China. Parasite.

[32] Corsi G.I., Tichkule S., Sannella A.R., Vatta P., Asnicar F., Segata N., Jex A.R., van Oosterhout C., Cacciò S.M. (2023). Recent genetic exchanges and admixture shape the genome and population structure of the zoonotic pathogen *Cryptosporidium* parvum. Mol. Ecol..

[33] Agyabeng-Dadzie F., Xiao R., Kissinger J.C. (2024). *Cryptosporidium* genomics—Current understanding, advances, and applications. Curr. Trop. Med. Rep..

[34] Feng Y., Ryan U., Xiao L. (2018). Genetic diversity and population structure of *Cryptosporidium*. Trends Parasitol..

[35] Keidel J., Daugschies A. (2013). Integration of halofuginone lactate treatment and disinfection with p-chloro-m-cresol to control natural cryptosporidiosis in calves. Vet. Parasitol..

[36] Burnet J.B., Penny C., Ogorzaly L., Cauchie H.M. (2014). Spatial and temporal distribution of *Cryptosporidium* and Giardia in a drinking water resource: Implications for monitoring and risk assessment. Sci. Total Environ..

[37] Zahedi A., Paparini A., Jian F., Robertson I., Ryan U. (2015). Public health significance of zoonotic *Cryptosporidium* species in wildlife: Critical insights into better drinking water management. Int. J. Parasitol. Parasites Wildl..

